# Chronic cough and obstructive sleep apnea in a community-based pulmonary practice

**DOI:** 10.1186/1745-9974-6-2

**Published:** 2010-04-15

**Authors:** Krishna M Sundar, Sarah E Daly, Michael J Pearce, William T Alward

**Affiliations:** 1 Intermountain Utah Valley Pulmonary Clinic, 1055N, 300W, Provo, UT 84604, USA; 2 Merrill Gappmayer Family Medicine Center, 475W, 940N, Provo, UT 84604, USA; 3 Pulmonary Division, Department of Medicine, University of Utah, 26N, 1900E, Salt Lake City, UT 84132, USA

## Abstract

**Background:**

Recent reports suggest an association between unexplained chronic cough and obstructive sleep apnea (OSA). Current guidelines provide an empiric integrative approach to the management of chronic cough, particularly for etiologies of gastroesophageal reflux (GERD), upper airway cough syndrome (UACS) and cough variant asthma (CVA) but do not provide any recommendations regarding testing for OSA. This study was done to evaluate the prevalence of OSA in patients referred for chronic cough and examine the impact of treating OSA in resolution of chronic cough.

**Methods:**

A retrospective review of chronic cough patients seen over a four-year period in a community-based pulmonary practice was done. Patients with abnormal chest radiographs, abnormal pulmonary function tests, history of known parenchymal lung disease, and inadequate followup were excluded. Clinical data, treatments provided and degree of resolution of cough was evaluated based on chart review. Specifically, diagnostic testing for OSA and impact of management of OSA on chronic cough was assessed.

**Results:**

75 patients with isolated chronic cough were identified. 44/75 had single etiologies for cough (GERD 37%, UACS 12%, CVA 8%). 31/75 had multiple etiologies for their chronic cough (GERD-UACS 31%, GERD-CVA 5%, UACS-CVA 3%, GERD-UACS-CVA 3%). 31% patients underwent further diagnostic testing to evaluate for UACS, GERD and CVA. Specific testing for OSA was carried out in 38/75 (51%) patients and 33/75 (44%) were found to have obstructive sleep apnea. 93% of the patients that had interventions to optimize their sleep-disordered breathing had improvement in their cough.

**Conclusions:**

OSA is a common finding in patients with chronic cough, even when another cause of cough has been identified. CPAP therapy in combination with other specific therapy for cough leads to a reduction in cough severity. Sleep apnea evaluation and therapy needs to considered early during the management of chronic cough and as a part of the diagnostic workup for chronic cough.

## Background

The revised ACCP guidelines provide a step-wise approach for managing patients with chronic cough [1]. These guidelines recommend basing the etiology of chronic cough upon clinical opinions derived from historical information and therapeutic interventions [1]. Considerable variations therefore result in the management of chronic cough. Variations in management also stem from the diagnostic workup used to ascertain the cause of cough [2] and also from the occurrences of multiple etiologies of chronic cough [3]. Recent reports have suggested an association between chronic cough and obstructive sleep apnea (OSA) [4]. There is also evidence that treatment of sleep apnea can improve chronic cough [5]. Despite the lack of any specific guidelines on testing for OSA in patients with chronic cough [6,7], the impact of treatment of OSA is being noted in community-based pulmonary practices where chronic cough is most frequently encountered.

This study was undertaken to evaluate current strategies in approaches to chronic cough in non-smokers without known parenchymal lung disease in a large community-based pulmonary clinic. Besides evaluating treatment regimens and diagnostic testing, the impact of diagnosis and treatment of sleep apnea on the course of chronic cough was also assessed.

## Methods

A retrospective review of medical records of patients seen in the Utah Valley Pulmonary Clinics in Provo and American Fork between 2005 and 2009 was done. Charts with diagnoses of "cough" and "bronchitis" were reviewed for cough lasting longer than 8 weeks. Since this study was confined to the evaluation of chronic cough in non-smokers without parenchymal lung disease, patients with abnormal chest X-rays, any prior smoking history, history of asthma requiring maintenance therapy, history of chronic parenchymal lung disease were excluded. Also patients that were not compliant with follow-up visits were excluded. Patients with only "normal" spirometry were included. Pulmonary function tests were conducted and interpreted based on the Intermountain Thoracic Society standards [8].

Following above exclusions, 75 patient records were identified and reviewed for clinical data, diagnostic workup and therapeutic interventions. Clinical data obtained included demographic information, cough duration, comorbidities, etiologies for chronic cough, treatments provided and ancillary laboratory, radiological, and physiological workup. Patient records were specifically reviewed for mention of details regarding concomitant or pre-existing evaluation for OSA. Details regarding sleep history, physical exam pertinent to sleep apnea (pharyngeal crowding, neck circumference more than 17" in males and 16" in females, moderate to morbid obesity) were looked for.

Polysomnography was done based upon pulmonologist's decision to pursue testing for OSA based upon findings of sleep history, physical exam consistent with a possibility of sleep disordered breathing and results of overnight oximetry. For patients undergoing polysomnography, the severity of sleep apnea was estimated based on the calculated apnea-hypopnea index [9]. Treatments and effects of therapy for chronic cough at the initial and following visits were reviewed. Improvements in cough were ascertained based on self-reported assessments of cough during followup visits.

Waiver of consent for the study was obtained from the Intermountain Office of Research. Part of the study findings were presented in an abstract form during CHEST 2009, San Diego, USA [10].

## Results

Patient records were reviewed from 8 different American Board certified pulmonologists. The number of included patients varied from 1 to 23 patients per provider, with a mean of 9. Patient characteristics, duration of cough, body mass index and co-morbidities are as shown in Table 1. Out of 75 patients, 44 patients had a single diagnosis for chronic cough at the time of the first visit with gastro-esophageal reflux disease (GERD) being the most common etiology (37%) followed by upper airway cough syndrome (UACS) (12%) and cough variant asthma (CVA) (8%) (Table 1). One patient was diagnosed and treated only for OSA. 31/75 (41%) patients had multiple diagnoses for chronic cough with the combination of GERD-UACS being the commonest followed by GERD-CVA and UACS-CVA (Table 1). Two patients received therapy for all three causes - UACS, GERD and CVA at first visit.

**Table 1:** Patient demographics, comorbidities and etiology of chronic cough.

Patient characteristics	N = 75
Age in years (mean ± SD)	57 (± 14)

Female:Male ratio	1.5

BMI kg/m^2 ^(mean ± SD)	
Overall	32 (± 8)
Male	31 (± 5)
Female	33 (± 9)

Duration of cough in weeks (mean ± SD)	
Overall	127 (± 274)
Male	55 (± 101)
Female	175 (± 337)

Comorbidities	
Hypertension	25 (33%)
Diabetes mellitus	9 (12%)
Rheumatoid arthritis	1 (1%)
Known sleep apnea	13 (17%)
Coronary artery disease	1 (1%)
ACE-I therapy	10 (13%)

Single diagnoses for cough	43/75 (57%)
GERD	28 (37%)
UACS	9 (12%)
CVA	6 (8%)

Multiple diagnoses for cough	31/75 (41%)
GERD & UACS	23 (31%)
UACS & CVA	2 (3%)
GERD & CVA	4 (5%)
UACS, GERD & CVA	2 (2%)

Abbreviations: BMI - Body mass index, ACE-I - Angiotensin converting enzyme inhibitors.

GERD was the commonest etiology for chronic cough (irrespective of whether diagnosis was made as part of single or multiple etiologies) followed by UACS and then CVA. Proportion of patients with diagnoses of GERD, UACS and CVA (single or multiple diagnoses of cough) were 76%, 48% and 19% respectively. In 39% of patients, there was a history of an upper or lower respiratory tract infection at the onset of cough although this occurred more than 8 weeks before the patient presented to the pulmonary clinic. 5/75 patients had stoppage of angiotensin-converting enzyme inhibitors as a part of their management of chronic cough.

The investigative workup for these patients is detailed in Table 2. Two patients underwent methacholine challenge testing with one test demonstrating bronchial hyperreactivity. All chest CT scans performed were normal. Sinus radiographs or CT scans were ordered in 10 patients with 3 showing evidence of sinusitis (Table 2). ENT referrals were made in 4 patients. Two patients underwent upper gastrointestinal endoscopy with one undergoing 24 hour pH monitoring (Table 2).

**Table 2:** Investigative workup for etiology of chronic cough and sleep-apnea specific workup in patients with chronic cough

INVESTIGATIONS PERFORMED	N (%)
Pulmonary Function Tests	
Spirometry	70/75 (93%)
Diffusion capacity	60/75 (80%)
Lung volumes	44/75 (59%)
Methacholine challenge testing	2/75 (3%)
Six-minute walk test	1/75 (1%)

Radiologic studies	
Chest X-ray	54/75 (72%)
Chest CT scan	10/75 (13%)
Sinus imaging	10/75 (13%)

Endoscopic studies	
Bronchoscopy	0/75
Upper GI endoscopy	2/75 (3%)
24 hour pH monitoring	1/75 (1%)
(Bravo* pH probe)	

Laboratory studies	5/75 (7%)

**SLEEP APNEA RELATED WORKUP**	

Sleep history obtained	41/75 (55%)
Abnormal	36/75 (48%)

Screening overnight oximetry	6/75 (8%)
Abnormal	6/75 (8%)

Polysomnography	38/75 (51%)
Sleep disordered breathing	33/75 (44%)
No OSA (AHI < 5)	4/75 (5%)
Mild OSA (AHI 6-15)	6/75 (8%)
Moderate OSA (AHI 16-30)	6/75 (8%)
Severe OSA (AHI >31)	14/75 (19%)
Periodic limb movement disorder	1/75 (1%)
Sleep efficiency (mean)	89%
Arousal index (mean)	17
Oxygen saturation (mean)	91%
Lowest oxygen saturation (mean)	78%

Abbreviations: OSA - Obstructive sleep apnea; AHI - Apnea hypopnea index.

* Bravo pH capsule with delivery system (Medtronic, Inc. Minneapolis, MN, USA)

67 patients in this study came as referrals from primary care providers (5 patients were self-referred to clinic). Two patients were referred from an ENT specialist and 1 from a gastroenterologist. Most if not all had been tried on multiple previous therapies including those for GERD, CVA and UACS. Despite this all patients were tried on therapeutic interventions based on the clinical impression of the treating pulmonologist. No patient underwent additional workup beyond chest X-rays and PFTs at the time of the initial visit. Initial and subsequent therapies were guided entirely by pulmonologist's empiric diagnosis of etiology of cough and therapeutic responses to rendered therapies. Although this approach broadly followed the outlines of the pathway for the management of chronic cough in the ACCP guidelines, there were variations from this pathway based on intention to pursue therapy based upon the perceived etiology. Percentage of improvement with different initial therapies for GERD, UACS and CVA was 82%, 56%, 83% respectively. In the groups with multiple diagnoses, initial therapies were successful in 78% of the UACS-GERD group, 50% of the GERD-CVA group and 100% of the UACS-GERD-CVA and UACS-CVA groups. Inhaled steroid therapy was done in 19% of patients and oral steroids were given in 4% of patients. 12 patients received empiric macrolide therapy in conjunction with other therapies that improved cough in 7 patients. Significant variations were noted in the proportion of patients treated for GERD, UACS and CVA between different providers.

A sleep history was elicited in 55% of the patients (Table 2). This included history of duration of sleep, sleep quality, daytime somnolescence, history of snoring and apneic spells. The decision to elicit history pertinent to the diagnosis of OSA varied amongst providers. A sleep history was consistently elicited in pulmonologists who were American Board certified in Sleep Medicine as well. Similarly details regarding historical aspects pertaining to OSA were variable. All six patients that underwent screening oximetry had abnormal studies. 12 patients had previously known OSA that was inadequately treated out of which 3 patients were not on any CPAP due to non-compliance with previously tried CPAP therapy. 34/38 patients had abnormal polysomnographies with 33 being diagnostic for OSA. Out of these 33 patients, 16 patients had initiation of CPAP therapy and 11 patients had retitration of their CPAP therapy. Improvement in cough was noted in 25/27 (93%) patients that had initiation of new CPAP therapy or re-titration to optimal CPAP pressures. CPAP therapy was initiated or re-titrated in 18/27 of patients following the first visit, 6/27 following the second visit and 3/27 of patients thereafter. Patient characteristics, duration of cough, concomitant diagnoses, and comorbidities of patients who were diagnosed with OSA during evaluation for chronic cough is shown in Table 3.

**Table 3:** Characteristics of patients diagnosed with OSA.

Patient characteristic	N = 33
Age (mean ± SD)	57 (± 13)

Female:Male ratio	1.3

BMI kg/m^2 ^(mean ± SD)	
Overall	35 (± 7)
Male	33(± 4)
Female	36 (± 8)

Duration of cough in weeks (mean ± SD)	
Overall	88 (± 262)
Male	23 (± 26)
Female	136 (± 341)

Comorbidities	
Hypertension	12 (37%)
Diabetes mellitus	7 (21%)
Known sleep apnea	12 (37%)
Coronary artery disease	1 (3%)
ACE-I therapy	6 (18%)

Single diagnoses for cough	
GERD	17/33 (52%)
UACS	2/33 (9%)
CVA	0

Multiple diagnoses for cough	
GERD & UACS	10/33 (30%)
UACS & CVA	0
GERD & CVA	1/33 (3%)
UACS, GERD & CVA	2/33 (6%)

Abbreviations: OSA - Obstructive sleep apnea; BMI - Body mass index; ACE-I - Angiotensin converting enzyme inhibitors.

## Discussion

The development of guidelines for evaluation and management of chronic cough represents a major milestone in the history of treatment of this common health problem [1]. Chronic cough accounts for 3.6% of outpatient physician visits in the US and is the commonest complaint for which medical attention is sought in the US [11].

Current guidelines emphasize empirical management of GERD, UACS and CVA depending on historical information gathered in favor of these diagnoses. This is based on the fact that a number of studies have consistently shown that UACS, GERD and CVA account for the majority of cases of chronic cough in the nonsmoker [12,13]. However, there is no understanding of the pathobiologic mechanisms by which these conditions lead to cough. Neither is there a defined pathological substrate that triggers cough from these conditions. This has led to difficulty in associating the results of investigative testing for UACS, GERD and CVA with the occurrence of cough. In addition, the common occurrence of these predisposing conditions in chronic cough patients and the lack of reliable tests to link GERD and UACS to cough results in therapeutic interventions being the mainstay for the diagnosis and resolution of the cough.

This study explores current approaches towards chronic cough in community-based pulmonologists from a single center in the United States. There has been a paucity of studies from North America on chronic cough evaluating current diagnostic and therapeutic trends over the last decade. This retrospective study evaluates management patterns of chronic cough over a time period overlapping and following the revised ACCP guidelines. Not surprisingly, it continues to show the same preponderance of etiologic diagnoses, namely GERD, UACS and CVA in patients with chronic cough and a tendency towards treating multiple etiological diagnoses during the initial visit. As reflected in the guidelines, the etiological diagnoses for chronic cough were based on therapeutic interventions despite the fact that a number of these referred patients underwent similar therapeutic interventions prior to evaluation by the pulmonologist.

The extent of therapeutic testing for chronic cough has been debated upon [14]. In this study, invasive testing for GERD, non-acid reflux disease, abnormal esophageal motility and testing for sputum eosinophilia was limited or lacking. The lack of a standardized protocol for evaluating sputum eosinophilia resulted in empiric therapy for CVA in a number of patients. Testing for OSA in patients with chronic cough has been recently recommended [5]. OSA is a common condition increasing in prevalence with age and body mass index [15] and therefore, likely to occur in a significant proportion of patients with chronic cough. Even though chronic cough has been reported to be a presenting symptom of OSA, no large prospective studies evaluating for OSA in chronic cough patients exist.

A major finding of this retrospective study was the impact of concomitant evaluation and treatment for OSA. OSA has been reported in prior case reports of chronic cough and one case series of four patients that resolved their cough with treatment for OSA [16,17]. In our current study, 44% patients with chronic cough were found to have OSA and following optimization of nocturnal positive pressure therapy, improvement or resolution of cough was noted in 93% of the patients. Since therapy for OSA was done in conjunction with other therapies for chronic cough in all but one patient, it is not clear to what degree the treatment for OSA had impact on the resolution of chronic cough. Despite this, the evaluation for OSA in the management of chronic cough requires important consideration given the increasing number of reports reporting improvement in cough with treatment of OSA. OSA can lead to or has been associated with GERD, asthma symptoms and upper respiratory complaints, all of which underlie the "pathogenic triad" leading to more than 95% of chronic cough [18]. OSA has been shown to be associated with airway inflammation that can contribute to chronic cough. In a study performed in Sweden, the number of patients with chronic bronchitic symptoms that were found to have sleep-disordered breathing was up to 14-29% [19]. Other studies on patients with OSA have shown an increase in exhaled nitric oxide values and other markers of inflammation on sputum analyses [20,21]. A number of OSA patients can present with bronchitic symptoms and demonstrate bronchial hyperreactivity [22,23]. Treatment of OSA has been shown to improve other known disorders of airway inflammation, especially asthma and COPD. Whether this is as a result of lessening gastroesophageal reflux that is common with OSA [24] or due to improvement in airway inflammation is unknown.

As compared to other series, the diagnosis of unexplained cough was not given to any of our patients. A significant incidence of unexplained cough has been noted in different series [25]. Interestingly the profile of patients reported for unexplained cough patients fits in with those patients in our series that improved with specific therapy for OSA [25]. A number of these patients start out with a post-infectious cough that fails to resolve despite multiple therapies directed at GERD, UACS or CVA. Whether OSA can perpetuate cough by impairing resolution in patients with acute bronchitis needs to be evaluated in future studies. OSA can potentially contribute to abnormal esophageal motility [26] and an enhanced cough reflex [16], both of which have been shown to contribute to or perpetuate cough.

This study is limited by a retrospective design, non-standardized protocol and data collection with only 55% of subjects being screened for OSA. Despite this a significant number of patients were found to have OSA. Whether this high prevalence of OSA in our chronic cough population is due to some kind of referral bias or due to a higher body mass index of patients is not clear. The majority of cough patients came from primary providers who considered possible etiologies for chronic cough as outlined in the ACCP guidelines but failed to ascribe any relationship between the possibility of sleep-disordered breathing and the cough. Henceforth a number of these patients were not evaluated for possibility of sleep-disordered breathing or if they had known OSA, the possibility of inadequately treated OSA contributing to cough was not entertained. Although only half the patients underwent workup for OSA and this was expected to reduce the estimate of OSA-cough in this population, the prevalence of OSA encountered in this study is nevertheless very high (44%). The majority of patients undergoing sleep apnea-related workup had an elevated BMI that makes obesity a confounding factor in this study purporting a link between OSA and chronic cough. Ascribing a relationship between chronic cough and OSA in obese subjects may also carry an overlap bias given the common occurrence of these problems and the linear relationship between obesity and OSA. However, the majority of obese patients in this study improved their cough following CPAP therapy and since resolution of cough remains the *sine qua non *for the diagnosis of the etiology of cough [6], further prospective studies researching the link between chronic cough and OSA will have to be designed factoring in the contribution of obesity. In addition, treatment for OSA can improve the contribution from multiple etiologies especially GERD that improves with the treatment of OSA. This study was also confined to the evaluation of cough in non-smokers without parenchymal lung disease. A number of recent studies have shown a high prevalence of OSA in patients with interstitial or airway lung disease [27,28]. Treating OSA early on in patients with parenchymal lung disease may not only offer the potential of impacting the course of the underlying lung disease but also the potential for amelioration of the cough seen in these disorders [29].

A small number of patients in this study received macrolides that were effective in 70% of those treated. Azithromycin used for up to 12 days improved cough in subsets of patients that also received PPIs. Macrolides have been shown to have beneficial effects on lower respiratory tract inflammation in a number of diseases ranging from asthma to post-transplant bronchiolitis [30]. Whether resolution in cough following macrolide therapy is due to its salutary effects on lower-respiratory tract inflammation or due to effects on sinus inflammation needs to be proven.

## Conclusions

This retrospective evaluation of management of patients with chronic cough in nonsmokers found that GERD, UACS and CVA continued to be the commonest etiologies for chronic cough. A significant proportion of patients had multiple etiologies for their chronic cough and specific diagnostic workup was limited. Clinicians primarily relied on the results of therapeutic interventions in cases with single or multiple etiologies for chronic cough. A number of patients improved with therapy of OSA that was given in conjunction with other therapies for chronic cough. The impact of OSA in occurrence and perpetuation of chronic cough needs to be evaluated prospectively in future studies of chronic cough.

## Abbreviations

OSA: Obstructive sleep apnea; GERD: Gastroesophageal reflux disease; UACS: Upper airway cough syndrome; CVA: Cough variant asthma.

## Conflicts of interests

The authors declare that they have no competing interests.

## Authors' contributions

KS was involved in study design and planning, review of data and manuscript preparation; SD in collection and organization of data and manuscript preparation; MP & WA in review of data and drafting the manuscript. All authors read and approve the final manuscript.
